# Lightweight Multimodal Domain Generic Person Reidentification Metric for Person-Following Robots

**DOI:** 10.3390/s23020813

**Published:** 2023-01-10

**Authors:** Muhammad Adnan Syed, Yongsheng Ou, Tao Li, Guolai Jiang

**Affiliations:** 1 Shenzhen Institutes of Advanced Technology, Chinese Academy of Sciences, Shenzhen 518055, China; 2 Konka R&D Department, Konka Group Co., Ltd., Shenzhen 518053, China; 3 Guangdong Provincial Key Laboratory of Robotics and Intelligent System, Shenzhen 518055, China

**Keywords:** person re-identification, impostor resisting metric, multi-modal re-identification metric, lightweight domain generic metric, part-wise attention learning

## Abstract

Recently, person-following robots have been increasingly used in many real-world applications, and they require robust and accurate person identification for tracking. Recent works proposed to use re-identification metrics for identification of the target person; however, these metrics suffer due to poor generalization, and due to impostors in nonlinear multi-modal world. This work learns a domain generic person re-identification to resolve real-world challenges and to identify the target person undergoing appearance changes when moving across different indoor and outdoor environments or domains. Our generic metric takes advantage of novel attention mechanism to learn deep cross-representations to address pose, viewpoint, and illumination variations, as well as jointly tackling impostors and style variations the target person randomly undergoes in various indoor and outdoor domains; thus, our generic metric attains higher recognition accuracy of target person identification in complex multi-modal open-set world, and attains 80.73% and 64.44% Rank-1 identification in multi-modal close-set PRID and VIPeR domains, respectively.

## 1. Introduction

With the advent of deep learning, human–robot interaction (HCI) is increasing rapidly in many applications. A robot following a person is one such application [1,2], where the person-following robots assist humans in elderly assistance and healthcare, work as service robots in industrial use, and also serve as autonomous carts in shopping malls.

**Figure 1:**
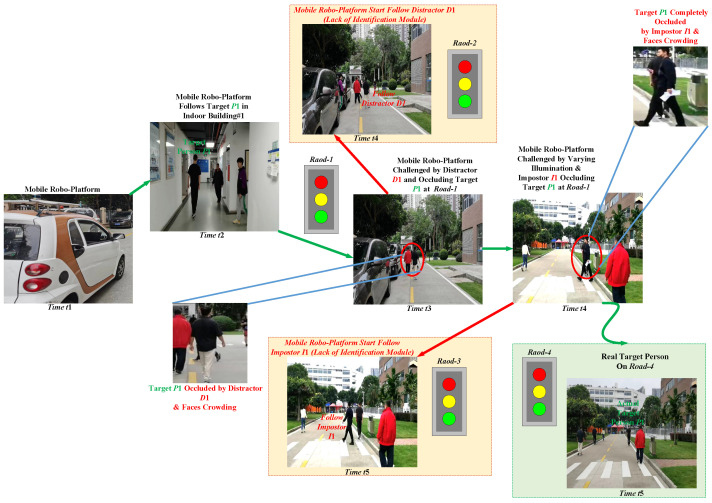
Person-follow robot scenario in outdoor world. The Mobile Roboplatform is Distracted in the Real Multi-Modal Open-Set World During Tracking Target P1.

Clearly, for all the applications above, it is required to track the person, and for tracking the person, the fundamental step is to first accurately identify the target person *P*1 shown in Figure 1, and then robustly track the target *P*1 in real-world. However, the dynamic real world is highly nonlinear and multimodal where the appearance of target person *P*1 shown in Figure 1 drastically varies from indoor home environment or domain to outdoor domain, such as walking across Road1, Road2, and Road3 in Figure 1, as well as across different outdoor domains, such as shopping mall or airport due to continually varying styles, illumination, poses, and viewpoints. In Figure 1, it can also be noted that the real world is also crowded where the target person *P*1 is also either occluded by other persons, say occluded by distractor *D*1 at time *t*2 on walking across Road1, or is also occluded by impostor *I*1 at time *t*4. Thus, in the real world in Figure 1, due to occlusion and nearby impostors, tracking the target person *P*1 is a very difficult task, hence, state-of-the-art trackers [3,4,5,6] could lose the tracking of target person *P*1, and either wrongly start following distractor *D*1 at time *t*4, or wrongly start following an impostor person *I*1 at time *t*5, as shown in red rectangles in Figure 1. The real target person *P*1 is walking on Road1 at time *t*4, and on Road4 at time *t*5, respectively. Therefore, robustly tracking the target person *P*1 in the nonlinear open world is still an unsolved problem, and it requires a robust target person identification in real time to reliably follow the person *P*1 in the real world.

In past, several works have addressed target person tracking and following. These past works use Laser Range Finder (LRF) [7,8], stereo camera [9], and RGB-D sensors [10] to track and follow the person. These trackers [7,8,9,10] have successfully addressed the person following problem, but these methods still get distracted in nonlinear and noisy outdoor environments due to occlusion, whereas these methods also lack the ability to reliably discriminate the target against similar-looking distractors in the outdoor world. Recently, deep visual trackers [11] have also learned to overcome the problem of reliably tracking the person in noisy environments; however, these deep visual trackers have still lacking to specifically address the target person identification problem during tracking and following. Visual trackers [11] are thereby still prone to impostors, and appearance changes the target person *P*1 undergoing in different nonlinear indoor and outdoor environments or domains.

Therefore, to overcome these shortcomings in visual trackers [11], recently, reidentification metrics have been learned and integrated with visual trackers [12] to follow the target person [1,2]. These reidentification metrics are learned by matching color-histograms and gait features [1,13], as well as extracting deep CNN features to learn deep similarity metrics [2,3,14,15,16]. However, the reidentification metrics in the present works [2,3,14,15,16] are all learned assuming the naïve world, i.e., it is assumed that the outside world is close-set, unimodal (it is assumed the robot only uses RGB sensor), and the target person *P*1 appearance remains unchanged across moving different domains. Due to these naïve assumptions, not only do the generalization capabilities of the learned reidentification metrics suffer largely in tracking the target in the outside world, but these metrics also get distracted due to the impostors observed across complex scenes and across nonlinear domains during tracking target *P*1, as shown in Figure 1. In Figure 1, at times *t*3 and *t*4 when the actual target *P*1 is completely occluded by distractor *D*1 and impostor *I*1 or if target *P*1 is completely moved out from the perception of mobile roboplatform in Figure 1, then the mobile roboplatform wrongly identifies a distractor *D*1 or impostor *I*1 as target and starts tracking the distractor *D*1 or impostor *I*3 at time *t*4 and *t*5, respectively.

Consequently, it is clear that to robustly track the target person *P*1 across different nonlinear indoor and outdoor environments, improvement of the trackers and integration of robust yet generic reidentification metrics in tracking are needed. The learned robust and generic reidentification metric, thus, identifies the target person in each consecutive image frame and thereby improves the tracking of the target while largely preventing the tracker to wrongly follow the distractors or impostors. Therefore, in our work, we have learned a lightweight domain generic reidentification metric, referred as $M_{G}$, for following the target in outside world. Furthermore, a lightweight novel part-attention learning method is also proposed to accurately identify the target *P*1 across different nonlinear domains, as well as to further improve the tracker to reliably track the target *P*1. The purpose of the lightweight metric is to run the metric on the compact robotic platform, while domain generic metric is learned to tackle (i) the appearance and style changes of target *P*1 across different domains, (ii) to tackle impostors of *P*1 in the scenes, and (iii) to reliably recapture the target person *P*1 again using the novel attention features, if target *P*1 is lost due to occlusion or completely moved out from the robot perception during tracking. The generalization capability of the learned reidentification metric in our work is further improved in a way that our domain generic metric $M_{G}$ is learned under realistic open-set scenarios, i.e., it is assumed that the appearance of the target person *P*1 varies when *P*1 moves across different indoor and outdoor environments. Then, the novel proposed attention module extracts the attention features from each single body part of the target person *P*1 to learn the deep cross-representations among the different images of the target person *P*1 undergoing appearance changes due to varying styles and modals. Therefore, the learned cross-representations are used to jointly address the pose and occlusion and also used to reject the large number of impostors during identifying the target in outside world, consequently largely improving the tracking of the target in outdoor world. In last, our contributions are:

- A robust vision-based target reidentification metric is proposed for target tracking. Compared to previous reidentification metrics proposed to target tracking, our reidentification metric is cross-modal and can address the style changes across large number of varying environments.
- Our reidentification learns part attention features, and unlike past works, the attention features are more stable to style changes and more robust against impostors. This is because the attention maps in our work are learned locally for each individual part, while during attention learning, it also uses global contextual and semantic information of the individual part. The global contextual provide useful relations among parts, while semantic information provides structural cues.
- Furthermore, the proposed reidentification uses the cross-representation module to jointly address pose and viewpoint changes and learns discriminative cross-view representations to tackle a large number of impostors in the open-set world.
- Finally, the learned metric is learned for the purpose of target tracking; therefore, it is designed with a lightweight backbone, while it is generic to help tracking the target in different nonlinear environments.

## 2. Related Work

The aim of our work is to learn a reidentification metric to integrate with tracking. Therefore, in this section, we review the recent works learning the reidentification metric for target tracking. Furthermore, in this section, we also review the recent works learning the reidentification metric for target tracking on mobile robots, and in the last, we also review the state-of-the-art related work in person reidentification that learns robust person identification metrics. In the beginning, we first cover the present works learning the reidentification metric for the visual tracking purpose.

### 2.1. Reidentification Metrics in Visual Tracking

Here, we cover the related work that learns reidentification metric for visual tracking purpose. However, the most of the learned models are used for offline tracking purpose. In [17], Neeti et.al. learned a LSTM-based CNN tracker using person reidentification module. They have learned spatiotemporal features of the person for reidentification; however, during training, it is assumed that the real world is close-set and unimodal, and hence, the model performance could be challenged when deployed in the multimodal open-set world.

In another work in [18], tracking with person reidentification is learned, where the learned tracker tracks the target person in traffic scenes. Similarly, in [19], the authors follow the tracking-by-detection method, where the CNN-based feature matching is used to identify the target person in consecutive frames, and thus, the tracking of the target is done. The frameworks in [18,19] are simple, but these works require fine-tuning the reidentification metric every time for for every unseen domain, and thus have low generalization. Other works including [20,21] solve tracking in the multicamera network for smart city applications. [Edge Video] focuses to learn a lightweight reidentification metric to implement target tracking on edge devices, while Ref. [21] solves the problem of retracking the target after occlusion. Ref. [21] uses reidentification metric to reidentify the target when the target is recaptured again after the occlusion. Even [20,21] have good performance, however, ignored to solve reidentification problem in multimodal open-set world.

Furthermore, Ref. [22] also addressed the problem of target tracking in large scale scenario and proposed to learn an unsupervised reidentification metric for this purpose. The authors believed for the large-scale scenario that unsupervised reidentification requires no label of identities for training, and thus perform better than supervised metrics. Although the above works [17,18,19,20,21,22] used the learned reidentification metric for visual tracking, the methods are not intended for tracking the target on mobile robots. In order to track the target on mobile robots, lightweight and efficient reidentifications are needed; therefore, in the next subsection, we cover the recent works that specifically learn the reidentification metrics for tracking the target in real time and on mobile robots.

### 2.2. Reidentification Metrics for Visual Tracking on Mobile Robots

Here, we cover the recent state-of-the-art works learning reidentification metric for person identification and tracking for mobile robots. In [1] height, gait, and appearance features are used to learn an online person classifier to identify the target person to follow, while, Ref. [23] uses human pose estimation to detect the person indoors and then identify the target person using an appearance-features-based reidentification metric to follow. In addition, both [24] and [25] also use the appearance-features-based reidentification metric to detect and track the target person in the indoor environment. Although the method from [1,23,24,25] tracks well indoors, it lacks the ability to handle nonlinear style and appearance variations that the target person undergoes in outdoor world, and it is largely distracted due to impostors in the outdoor environment.

On the other hand, Ref. [15] also uses convolutional channel features to first identify the target person and then follow the identified target person using the mobile robot. Both [15] and [26] first use laser range finder to track the person position, then Ref. [15] learns the convolutional-channel-features-based classifier to verify the target to follow, whereas Ref. [26] uses monocular camera to perform appearance matching. In another work [2], an online person classifier is also learned to track the target person, but in the robot coordinate space. The authors believed tracking the person in robot coordinate space is more accurate than tracking in the real-world space. The methods [1,2,15] follow the person in both indoor and outdoor environments; however, the learned person classifiers in their works are not generic, while their works also fail to address resisting the impostors in the outdoor world.

Furthermore, some works have used depth sensing to track the position of target. Both [27] and [28] use kinect depth sensor to track the person position. The robot tracks the person, however, in the crowded environment; due to distractors, the accuracy of tracking is largely challenged in the outdoor setting due to the sensitivity of the Kinect sensor.

In other works, authors, e.g., those of [14], propose to track the target person using the Kalman filter. Once the bounding box of target person predicted, the state of Kalman filter is updated by identifying the target bounding box, and the target is then followed. In [3], another problem of tracking the target in uniform crowd environment is solved. Their method depends on accurate face identification because the target and other persons all have similar appearances in the scene. Even the method performs well, but the method has still not addressed generalization problem across all outdoor environments, as well as Ref. [14] can be distracted due to impostors, whereas Ref. [3] can be distracted in crowded environment due to poor depth sensing.

In addition, there are a few works in reidentification-based person tracking on mobile robots aim to track the person for social and virtual game environments. In [29], both depth and laser range finder sensors are used to track the person position, however, only in the indoor environment, whereas Ref. [30] track and follow the person for the virtual game environment. Even their methods can track the target, but their methods are optimal for close-set scenarios, whereas the real-world scenario is multimodal and open-set. Now, covering the recent works in reidentification-based person tracking on mobile robots, we now further explore the state-of-the-art metrics learned for real-world person reidentification and are covered in detail in the subsection below.

### 2.3. State-of-the-Art Reidentification Metrics

#### 2.3.1. Deep Metrics

Deep metric learning for Person ReID has been extensively studied in past works, e.g., [31,32]. These metrics, though, aim to address pose, viewpoint, occlusion, and misalignment of parts to attain high similarity; however, they underperform against unseen domains.

#### 2.3.2. Domain Adaptation Metrics

To improve poor generalization, the authors of a few works proposed unsupervised domain adaptation (UDA) [33,34]. UDA adapts the learned ReID metric from labeled source domains to unlabeled target domain, but it is still time-consuming due to collecting data and fine-tuning the metric for each new unseen target domain.

#### 2.3.3. Domain Generalizable Metrics

Therefore, recently, domain generalizable metrics have gained a great amount of attention in ReID [35,36,37,38]. Domain generic metrics are learned once and then directly applied for identification on previously unseen domains; however, existing metrics still ignore that the real world is multimodal and open-set, where the same person is seen in several different styles and modals. Therefore, it is desired that the generic metric in the real world (i) matches different Probe and Gallery images of the same person in different modals and styles and (ii) jointly addresses pose, viewpoint, and displacement of parts across views, while (iii) is also robust against impostors in open-world, and (iv) is lightweight, thus it can run on devices in the real world.

## 3. Methodology

**Figure 2:**
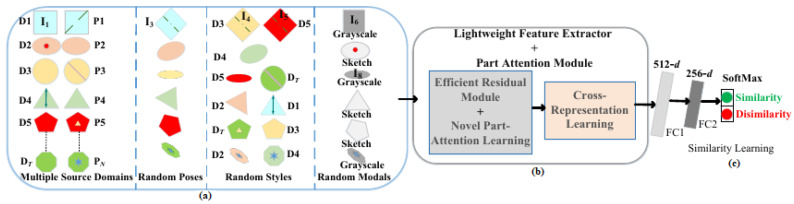
Framework of domain generic metric $M_{G}$. (**a**) Real Open-world Training Data. (**b**) Lightweight Features Extractor with Novel Attention and Cross-Representation. (**c**) Similarity Learning.

In this work, our aim is to learn person reidentification metric to identify the target person and to integrate the learned reidentification metric with pretrained state of the art visual tracker to track the target; therefore, in this section, we will cover the details of learning the cross-modal domain generic open-set person reidentification metric and will describe its complete framework as shown below in Figure 2. In Figure 2, first realistic training data from open-set multi-modal world is generated, as shown in Figure 2a. In Figure 2a, a large number of images of *N* different person identities, say person *P*1, *P*2, and $P_{N}$, as shown in Figure 2a, are taken from $D_{T}$ different source/camera domains.

Then, in the next step, a large number of images of each person identity are generated in different random poses. The purpose of generating images in different random poses for each different identity is to augment the training images of each person identity in different poses to learn its pose invariant features for identification. Next, in Figure 2a, taking all the original images of *N* persons and their generated images in random poses, we now randomly transfer different images of different persons into different random styles. This is done because in the real world, when tracking the person in the outdoor world, it could move across several different environments, and in each different environment, it could undergo nonlinear style variations; thus, in order to identify the target person in different environments and in different styles, it is needed to learn a style robust person reidentification metric, and therefore, the images of a person are generated in multiple styles to train the reidentification metric in such a way that it could match all the images of the same person in all the different random styles. Finally, in the real world, it is also needed to obtain a cross-modal reidentification metric because it is possible that different mobile robot platforms use different imaging sensors, such as RGB and IR cameras. Therefore, in Figure 2a, to learn a cross-modal reidentification metric, our work takes different original images of *N* persons and takes their generated images in random poses and in random styles to randomly transform these different training images into different modals, say RGB, Grayscale, and Sketch modals. In our work, RGB images are transformed into Grayscale and Sketch modals due to the reason that in the real world, it is not necessary to always use an RGB sensor on a mobile robot; already, a large number of works have used IR modality [12]. However, a large number of public reidentification datasets have no IR images; therefore, we opted to transform RGB images into grayscale images. Sketch images are also generated to further improve the feature extraction power of the learned metric as well as to help in improving the cross-modality matching. After getting the realistic training data, a lightweight deep CNN backbone is then used to extract the features from the training images, as shown in Figure 2b. This deep CNN feature extraction backbone is designed using efficient residual module as shown in Figure 2b and is described in detail in Section 3.2.1. Furthermore, the feature extraction backbone also uses a novel part-attention module, as shown in Figure 2b, to extract the subtle features of the different individual parts. The details of novel part attention module are covered in Section 3.2.2. Next, using the learned attention features of different individual parts, our work learns cross-representations using the cross-representation learning module as shown in Figure 2b. The purpose of cross-representation learning is to minimize both the style and modality differences across cross-view features, which then feed to fully connected layers to learn the complex feature relationships to finally predict the similarity between the pair of images, as shown in Figure 2c.

Now, below in Section 3.1, we will cover the details of generating training data in the complex nonlinear multimodal open-set world.

### 3.1. Multimodal Open-World Training Data

**Figure 3:**
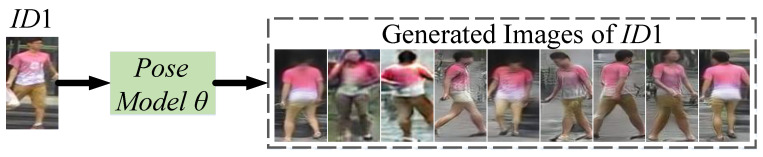
A Few Generated images of ID1 During training. Images are Generated using Pose Model θ [39].

Here, we describe the details of generating training data in nonlinear multimodal open-set world. The real world is nonlinear, multimodal, and open-set where a person can be seen across several different domains in different styles and can also experiences pose, viewpoint, and parts displacement. Therefore, to train a robust metric for the real world, it is first needed to obtain realistic world data for training. For this purpose, first, *N* number of different persons are randomly chosen from $D_{T}$ source domains, as shown in Figure 2a. In order to generate realistic open-world training data, in the first step, as shown in Figure 2a, images in random poses are generated for each person. Taking the image $I_{1}$ of person *P*1 seen in domain *D*1, as shown in Figure 2a, a new image instance $I_{3}$ for *P*1 in random pose $\theta_{p}$ is generated as:

(1) $$I_{3}=\theta \left(I_{1},\theta_{p}\right),$$

here θ is pose generation model [39]. Now, Equation (1) is used to generate images in random poses for all the *N* IDs in training data (in our work, images are generated in 8 random poses), and a few generated images for ID1 are shown below in Figure 3. Getting images for all the *N* persons in random poses, our work now generates images in varying styles for all *N* IDs. In Figure 2a, two instances $I_{4}$ and $I_{5}$ for ID *P*1 are generated in the styles of domain *D*3 and *D*5, respectively, as:

(2) $$\left[I_{4},I_{5}\right]=G\left(\left[w_{G_{D_{3}}},w_{G_{D_{5}}}\right],I_{3}\right),$$

here $w_{G_{D_{3}}}$ and $w_{G_{D_{5}}}$ are the parameters of translation model *G* [33] for domains *D*3 and *D*5, respectively, and $I_{3}$ is the input image. In Equation (2), the purpose of generating images for *P*1 in varying styles [33,34] is to exploit the diverse and varying styles images of *P*1 to learn its style generic representation; thus, the learned generic metric could distinguish *P*1 seen in any random style in the open world. Hence, our work generates images of *P*1 (i) in varying styles in different disjoint views of the same domain *D*1 [34], (ii) in varying styles across random Re-ID domains [33], e.g., in styles of domains *D*3 and *D*5 as shown in Figure 2a, and in varying styles of random detection and recognition datasets, such as Imagenet [40]. Images in varying styles and poses are obtained for *N* persons; however, the real world is actually multimodal. Therefore, Grayscale image $I_{6}$ and Sketch image $I_{8}$ in Figure 2a for IDs *P*1 and *P*4 are respectively generated as:

(3) $$\begin{array}{c} I_{6}=\varphi \left(I_{1}\right),\\ I_{8}=\phi \left(I_{7}\right), \end{array}$$

here, function φ and ϕ convert RGB images $I_{1}$ and $I_{7}$ of *P*1 and *P*4 into Grayscale and Sketch modals, respectively. Now, in the next Section 3.2, we now describe the details of learning person features using the proposed novel part attention module.

### 3.2. Novel Part-Attention Feature Learning

Now, using the generated cross-modal open-world training data, here, in this section, we cover the details of features extraction and novel part attention module. In the first, as shown in Figure 2b, person features are extracted with efficient lightweight CNN backbone and then, as shown in Figure 2c, the similarity between extracted pair of part attention features are learned. However, before covering the learning of features extraction, in our methodology, we first cover the details and design process of lightweight CNN backbone shown in Figure 2b below in Section 3.2.1.

#### 3.2.1. Lightweight Backbone Design

**Figure 4:**
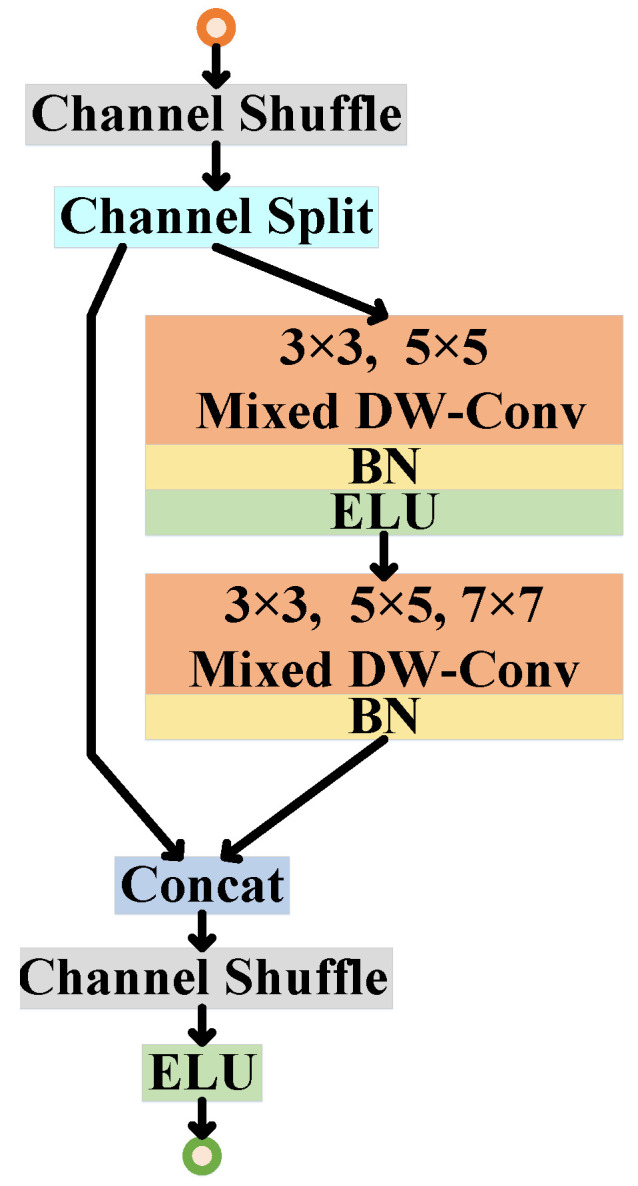
Our Efficient Residual Module Design.

Unlike [35,36,37,38], efficient residual module [40] is designed in Figure 4 to build a lightweight CNN backbone for learning cross-representations as shown in Figure 2b and learning the similarity between pair of learned features as shown in Figure 2c. Lightweight backbone as shown in Figure 2b is a Siamese network with 10-layers (details of layers are listed in Table 1), but for simplicity, only one stream is shown in Figure 2b. Each Convolution layer in Figure 2b is then realized with efficient residual module, where all convolutions are implemented as mixed depthwise separable convolutions [41] following Wider ResNet [42] strategy, i.e., the number of filters in each successive convolution layer are increased 2×times than previous convolution layer, thereby improving the features representation power with minimal computational cost. In addition, channel shuffle and channel split [43] are also used in efficient residual module in Figure 4 to enable information mixing across different filters and layers, thus further increasing diversity in features. After every convolution layer in Figure 4, Batch Normalization (BN) and ELU activation function are used for faster network convergence. Realizing the lightweight CNN backbone in Figure 2b, then, deep cross-representations are learned for each individual part of a person complimented with novel part-attention learning mechanism, as shown in Figure 2b.

#### 3.2.2. Novel Part Attention Cross-Representations

**Figure 5:**
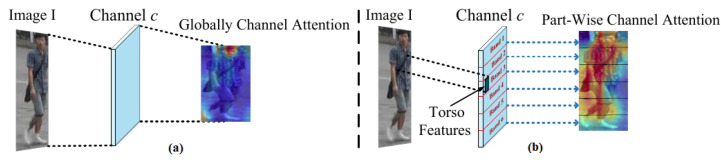
(**a**) Global channel attention [44,45]. (**b**) Our part-wise channel attention.

Attention learning has been proved promising in Re-ID; however, methods from past works [35,44,45] learn attention globally for the whole body as shown in Figure 5a, and thereby, certain valuable and unique features from different individual parts are loss. Therefore, we argue to learn attention features for each individual part, as shown in Figure 5b, to prevent from loss, as well as, highlight the unique cues of different parts.

**Figure 6:**
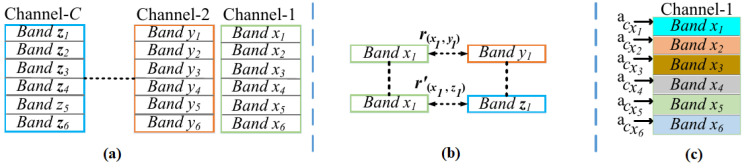
Novel channel attention learning for individual parts. (**a**) Dividing *C* channels into six bands; (**b**) Learning relations among bands; (**c**) Channel attention weight a’${}_{c}$.

Novel Channel Attention for Individual Parts: Taking input features *F*∈$R^{C\times H\times W}$, where *C* is the number of filters, while, H×W are feature spatial dimensions, channel attention for each individual part is then learned by horizontally dividing all the *C* filters into six spatial bands, as shown in Figure 6a, because each spatial band carries features of different part, as shown in Figure 5b, therefore, learning the attention for different corresponding bands, consequently, learns the attention weights for different corresponding parts. Hence, each single channel is partitioned into six horizontal bands, then the channel attention weight for each single band from each single channel is learned, but before the attention weight is learned it is first needed to capture the spatial, structural, and semantic relations of each band with its *C* neighboring bands, as shown in Figure 6b. Thus, similar to [44] relationships *r* and $r^{\prime }$ between bands $x_{1}$ and $y_{1}$, and between bands $x_{1}$ and $z_{1}$ are learned as:

(4) $$r_{\left(x_{1},y_{1}\right)}=\vartheta_{c}\left(f_{x_{1}}\right)\cdot \mu_{c}\left(f_{y_{1}}\right),$$

here, $f_{x_{1}}$ and $f_{y_{1}}$ are features of $x_{1}$ and $y_{1}$ with dimensions $R^{1\times \frac{H}{6}\times \frac{W}{6}}$, and the value $r_{\left(x_{1},y_{1}\right)}$ computed as dot product between embedding functions $\vartheta_{c}$ and $\mu_{c}$ [44]. Embedding functions $\vartheta_{c}$ and $\mu_{c}$ are implemented by first flattening features $f_{x_{1}}$ and $f_{y_{1}}$, then, apply 1×1 convolution followed by BN and ELU activation. Now, using Equation (4), all the *C* relations of band $x_{1}$ with all the *C* neighboring bands are then obtained to form the relation vector $r_{1}$ for $x_{1}$ as: $r_{1}$=${\left[r_{c_{\left(x_{1},:\right)}}\right]}_{c=1,...,C}$. Relation vector $r_{1}$ is then embed with features $f_{x_{1}}$ as:

(5) $$f_{x_{1}}^{\prime }=\left[{pool}_{Av}\left(\nu_{c}\left(f_{x_{1}}\right)\right),{pool}_{Mx}\left(\nu_{c}\left(f_{x_{1}}\right)\right),v_{c}\left(r_{1}\right)\right].$$

here ${pool}_{Av}$ and ${pool}_{Mx}$ are global average and max pooling operations. Embedding function $\nu_{c}$ first flattens features $f_{x_{1}}$, then, both $\nu_{c}$ and $v_{c}$ are implemented as 1×1 Conv followed by BN and ELU activation. Now, features $f_{x_{1}}^{\prime }$ are used to learn channel attention weight $a_{c_{x_{1}}}$, shown in Figure 6c, for band $x_{1}$ as:

(6) $$a_{c_{x_{1}}}=Sigmoid\left(W_{2}ELU\left(W_{1}f_{x_{1}}^{\prime }\right)\right)),$$

here, *W*1 and *W*2 are 1×1 Conv followed by BN. Now using Equations (4)–(6), first, all the six weights $a_{c_{x_{1}}}$, $a_{c_{x_{2}}}$, $a_{c_{x_{3}}}$, $a_{c_{x_{4}}}$, $a_{c_{x_{5}}}$, and $a_{c_{x_{6}}}$, shown in Figure 6c, for all the six bands in Channel-1 are computed, then, similarly, all the six weights for all the six bands in all the *C* channels are computed. Now, computing all the six weights for all the *C* channels, the six weights of each channel, e.g., Channel-1 weights shown in Figure 6c are taken and then each weight of each corresponding band is broadcasted similar to [45] over the spatial dimensions of each corresponding band to finally obtain channel attention $a_{c}^{\prime }$∈$R^{H\times W}$ for Channel-1. Following this, the channel attention $a_{c}^{\prime }$ for all the *C* channels are then obtained, and finally, the attention maps for all *C* channels are concatenated together to form matrix $A_{c}$ as: $A_{c}={\left[a_{c^{\prime }}^{\prime }\right]}_{c^{\prime }=1....C}$. Channel Attention features are now computed as:

(7) $$F_{a_{c}}=F⊗A_{c},$$

here ⊗ denotes elementwise multiplication [45] between weights $A_{c}$ and features *F*.

**Figure 7:**
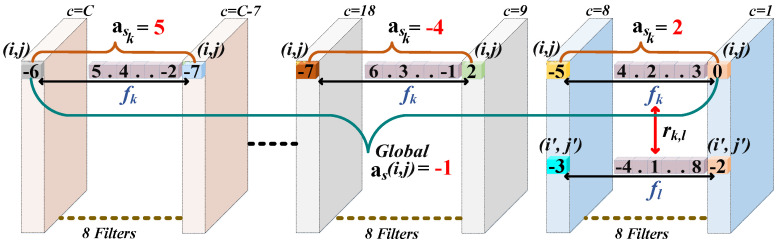
Novel Spatial Attention Learning for Individual Parts.

Novel Spatial Part Attention Features: Unlike past [35,44,45], our work learns spatial attention for pixel (*i*,*j*) for every 8 filters, i.e., weight $a_{s_{k}}$ for every 8 filters as shown in Figure 7. This is done to improve spatial attention while preventing the loss of vital patterns that are largely diminish when spatial attention for pixel (*i*,*j*) is learned globally over all the *C* filters, e.g., $a_{s}$(*i*,*j*) = −1 in Figure 7. Now, every time taking 8 filters, e.g., filters *c* = 1 to *c* = 8 in Figure 7, spatial attention $a_{s_{k}}$ for pixel (*i*,*j*) is learned by first learning the relations of pixel (*i*,*j*) with all the (H×W)-1 pixels in the corresponding 8 filters as:

(8) $$r_{k,l}=\vartheta_{s}\left(f_{k}\right)\cdot \mu_{s}\left(f_{l}\right),$$

here $f_{k}$ and $f_{l}$ are 8-dimension feature vectors of pixels (*i*,*j*) and ($i^{\prime }$,$j^{\prime }$) as shown in Figure 7, while, $r_{k,l}$ is the leaned relation, and embedding functions $\vartheta_{s}$ and $\mu_{s}$ are implemented as 1×1 spatial convolution followed by BN and ELU activation. Now, Equation (8) is used to learn all the H×W relations of vector $f_{k}$ of pixel (*i*,*j*) to form the relation vector $r_{k}$ as: $r_{k}$= ${\left[r_{r^{\prime }}\right]}_{r^{\prime }=1,...,H\times W}$, then, vector $r_{k}$ embed with vector $f_{k}$ to form feature $f_{k}^{\prime }$ as:

(9) $$f_{k}^{\prime }=\left[{pool}_{Av}\left(\nu_{s}\left(f_{k}\right)\right),{pool}_{Mx}\left(\nu_{s}\left(f_{k}\right)\right),v_{s}\left(r_{k}\right)\right].$$

here, in Equation (9), embedding functions $\nu_{s}$ and $v_{s}$ are learned as 1×1 Conv followed by BN and ELU activation. Now, the attention $a_{s_{k}}$ for pixel position (*i*,*j*) is learned as:

(10) $$a_{s_{k}}=Sigmoid\left(W_{2}ELU\left(W_{1}f_{k}^{\prime }\right)\right)),$$

here *W*1 and *W*2 are 1×1 Conv followed by BN. Now, Equations (8)–(10) are first used to learn the spatial weights $a_{s_{k}}$ for pixel (*i*,*j*) for every 8 filters, as shown in Figure 7, and then, similarly, Equations (8)–(10) are also used to learn the spatial weights $a_{s_{k}}$ for all the H×W pixels in every 8 filters. Getting the spatial weights $a_{s_{k}}$ for all the H×W pixels in all the *C* filters, first, the learned corresponding H×W weights for every corresponding 8 filters are broadcasted [45]over the spatial dimensions H×W, and then the attention maps $a_{s_{g}}$∈$R^{8\times H\times W}$ (here *g* = 1 to *C*/8) for every corresponding 8 filters are obtained. These attentions maps are then concatenated together to form spatial attention weights matrix $A_{s}$ for all *C* filters as: $A_{s}$∈$R^{C\times H\times W}$. Then, finally, spatial attention features $F_{a_{s}}$∈$R^{C\times H\times W}$ are obtained as:

(11) $$F_{a_{s}}=F_{a_{c}}⊗A_{s}.$$

### 3.3. Multi-Modal Open-Set Generic Metric

Using the attention features of each single part, cross-representation module [46] shown in Figure 2b now learns the cross-representations for pair of features $f_{q_{1}}$ and $f_{q_{2}}$ as:

(12) $$g\left(q_{1},q_{2}\right)=CRM\left(f_{q_{1}},f_{q_{2}}\right).$$

here, cross-representation module CRM in Equation (12) not only learns the complex nonlinear relationships between features $f_{q_{1}}$ and $f_{q_{2}}$ to minimize the existing domain, style, and modality gaps in different environments between positive pair ($q_{1}$, $q_{2}$), but, at the same time also addresses the pose, viewpoint, and spatial miss-alignment across views. For the given quadruplet $q_{1}$, $q_{2}$, $q_{3}$, $q_{4}$, all the cross-representations g(q1,q2), g(q1,q3), and g(q2,q4) are learned using Equation (12), and the representations are then sent to fully-connected layers FC1 and FC2 shown in Figure 2c with 2-dimension softmax classifier to learn similarity between ($q_{1}$, $q_{2}$). The learned similarity value is then used to compute the quadruplet loss [47] $L_{quad}$ as:

(13) Lquad=∑n=1N[g(q1,q2)2−g(q1,q3)2+α1]Lquad=+∑n=1N[g(q1,q2)2−g(q2,q4)2+α2],

here, *N* are total number of quadruplets, $\alpha_{1}$ = 1 and $\alpha_{2}$ = 0.3 are the margin values used, while $q_{3}$ and $q_{4}$ are the impostors of $q_{1}$ and $q_{2}$, and randomly seen in any domain in any style and modality, respectively.

## 4. Experiments and Analysis

### 4.1. Datasets and Data Augmentation

For training metric $M_{G}$ Market1501, DukeMTMC-reID, CUHK03, and CUHK02 are used as training source domains, while $M_{G}$ is comprehensively evaluated using domains VIPeR, PRID, GRID, and i-LIDS. For cross-modal evaluation, SYSU-MM01dataset used the following settings in [48]. In addition, random cropping, horizontal flipping, random rotation, color jittering, random contrast, brightness, and label smoothing regularization [49] are used in training to prevent $M_{G}$ from overfitting.

### 4.2. Implementation Details

Lightweight CNN backbone in Figure 2b is trained from scratch with randomly initialized weights [50] for 600 epochs on single 16G NVIDIA RTX 2080 Ti GPU. The during-training image resolution is 224×224, with the Adam optimizer used with initial learning rate 8 ×$10^{-5}$, mini-batch size 64, and weight decay 5 ×$10^{-4}$. All of the code was written in Pytorch.

### 4.3. Evaluation Metrics and Protocols

Unlike [35,36,37,38], $M_{G}$ is comprehensively evaluated in challenging multimodal, multistyle, close-set, and open-set scenarios. Cumulative Matching Characteristics (CMC) at Rank-1 and mean average precision (mAP) are the metrics used for all close-set experiments, while for all open-set experiments, true target rate (TTR) is measured against false target rate (FTR) [51] as a performance metric. All results obtained after averaging over 10 trials.

#### 4.3.1. Naïve Close-Set Scenario

**Figure 8:**
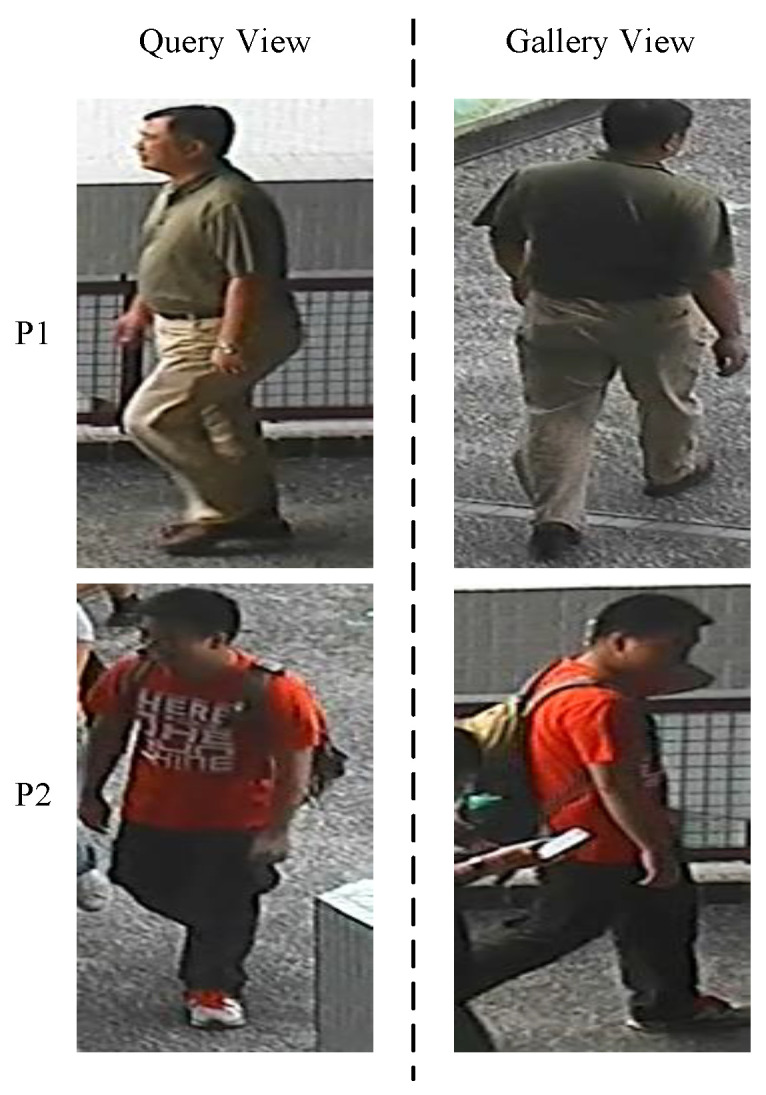
In Setting#1 it can be seen the matches of all Queries exist in the Gallery View, while the match for each person Seen in VIPeR domain is found from the Gallery of VIPeR domain domain.

Unlike [35,36,37,38], in this scenario, $M_{G}$ is evaluated in two different and difficult settings, in setting#1 and setting#2. In both settings, Probe/Gallery images splits are: VIPeR: 316/316; PRID: 100/649; GRID: 125/900; i-LIDS: 60/60, respectively. Here, VIPeR: 316/316 means there are 316 person identities in test Query view, and similarly, there 316 same person identities in the test Gallery view. In setting#1, matches of Queries observed in a corresponding domain, e.g., observed in CUHK-03 domain, are found from the Gallery view of the corresponding CUHK-03 domain only. Below in Figure 8, the testing scenario of setting#1 is shown.

**Figure 9:**
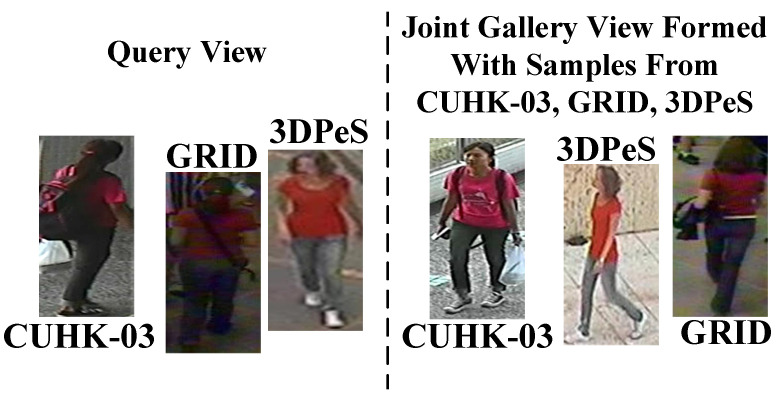
In Setting#2 the match for given Query image, the match for Query from GRID domain is found from the joint Gallery, where the Gallery contains images from all test domains, i.e., GRID, CUHK-03, and 3DPes.

On contrary, as shown in Figure 9, the matches of Queries in setting#2 are found from a joint Gallery containing Gallery images from all the test domains, i.e., VIPeR, PRID, GRID, and i-LIDS. Thus, in setting#2, $M_{G}$ is tested in more realistic and challenging scenario to find the matches of given Query by resisting large number of impostors from different outdoor environments. Identification results of $M_{G}$ in Setting#1 are summarized in Table 2, where $M_{G}$ attains 80.73%, 64.44%, and 88.99% Rank-1 identification on PRID, VIPeR, and i-LIDS, respectively.

Clearly, metric $M_{G}$ attains higher recognition than [35,36,37,38,44,52]; it is because $M_{G}$ learns cross-representations among different indoor and outdoor images of a person complimented with part-attention learning, where learned part-attention pays large focus on unique features from each different part, thus, $M_{G}$ jointly minimizes pose, viewpoint, and spatial displacement of parts, as well as jointly addresses style, modality, and domain gaps to resist large number of impostors in outdoor world.

**Figure 10:**
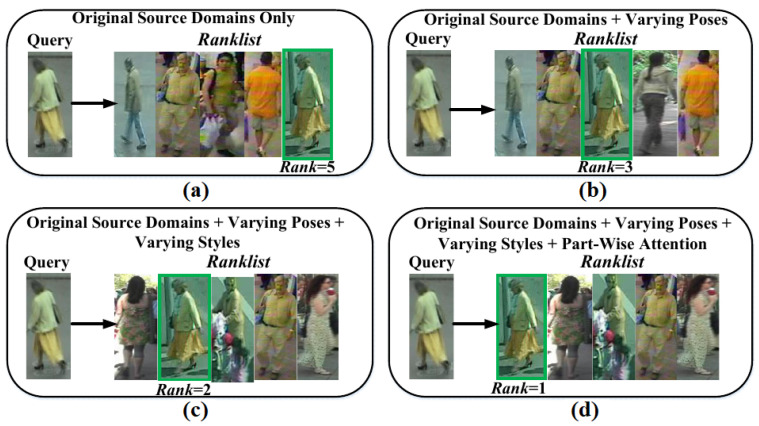
Retrieval results obtained in close-set setting#2 using different training data. (**a**) Training data only contains original image samples with no data augmentation; (**b**) Training data contains original image samples, and image samples are generated in different random poses too; (**c**) Training data contains original image samples, and image samples are generated in different random poses and in different random styles too; (**d**) Training data contains original image samples, and image samples are also generated in different random poses and styles, as well as, part-wise attention is learned to improve features power.

Furthermore, in setting#2, though, setting#2 is difficult than setting#1, but $M_{G}$ still surmounts the challenges in setting#2, and in Table 3, $M_{G}$ retrieves 77.89%, 52.64%, 61.92%, and 86.23% matches of the Queries at Rank-1 from the joint Gallery for test domains PRID, GRID, VIPeR, and i-LIDS, respectively. These results clearly reveal $M_{G}$ can inherently tackle pose, viewpoint, style, and modality transforms across both nonlinear indoor and outdoor environments, while, complemented with the part-attention mechanism, $M_{G}$ also learns cross-representations in a way to jointly address occlusion and misalignment of parts; therefore, in Figure 10d, $M_{G}$ declines the large number of impostors and improves the identification accuracy from Rank = 5 in Figure 10a to Rank = 1.

#### 4.3.2. Challenging Close-Set Scenario

**Figure 11:**
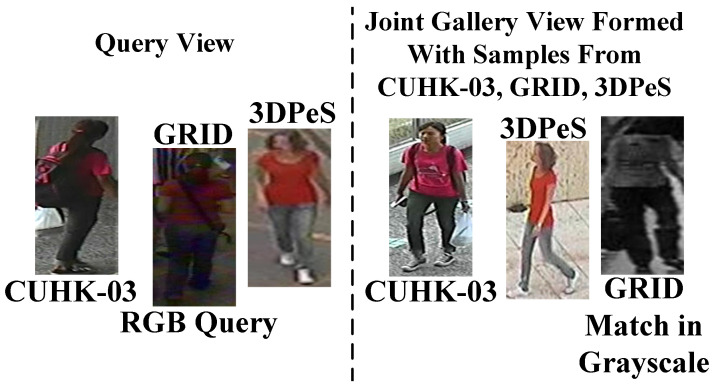
In the Challenging Close-Set Scenario, the Query image, the image of GRID domain could be seen in the RGB modalily (or different style), whereas its match in Gallery view has Grayscale modality.

The joint Gallery in setting#2 is very challenging; however, the target person being followed by robot when moving across different outdoor environments could undergo style, illumination, pose, and viewpoint changes in the real world. Therefore, to obtain a robust reidentification metric, $M_{G}$ is tested in the real-world environment where Probe-Gallery pairs could be seen in different modals [53] and in different styles [35]. The real-world testing scenario for Challenging Close-Set is shown below in Figure 11.

Therefore, during testing in the Challenging Close-Set Scenario, images in Query and in joint Gallery views are randomly transformed into different modals and styles, then, $M_{G}$ finds the matches from joint multimodal multistyle Gallery, and the results are given in Table 4.

**Figure 12:**
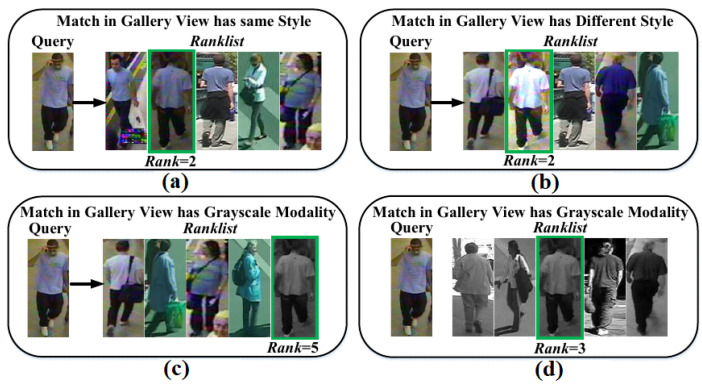
Retrieval results obtained in challenging close-set scenario. (**a**) Finding the match in Gallery view where the correct Gallery image has same style and modality with Query image; (**b**) Finding the match in Gallery view where the correct Gallery image has different style, but, same modality with Query image; (**c**) Finding the match in Gallery view where the correct Gallery image has different style, and different modality with Query image; (**d**) Finding the match in Gallery view where all the images in Gallery view has different style, and different modality with Query image.

In Table 4 $M_{G}$ attains 3.47% drop at Rank-1 accuracy than close-set setting#2. Therefore, the reasons for this drop are analyzed in retrieval results in Figure 12. In Figure 12a,b, it is evident that $M_{G}$ is robust against style changes and impostors and finds matches at Rank = 2; however, in Figure 12c $M_{G}$ lags in cross-modal matching due to impostors. Clearly, color images dominate intensity images, thus, to improve the multimodal recognition capability of metric $M_{G}$ in the outdoor environment, it is needed to optimally represent each person in RGB, Grayscale, and Sketch modals during training; hence, $M_{G}$ can resist a large number of multimodal impostors in outdoor environment.

**Figure 13:**
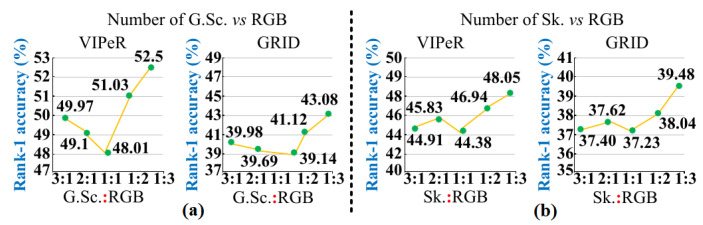
Rank-1 Result Comparison for Different Representation Ratios on VIPeR & GRID for (**a**) G.Sc. vs. RGB (**b**) Sk. vs. RGB.

Therefore, to find the optimal representation of persons in different modals, our work performed different experiments, and the results are shown in Figure 13. In Figure 13, for domains VIPeR and GRID, it is observed that $M_{G}$ declines a large number of impostors when a number of images for a large number of training persons have representation ratio 1:1:3 for Grayscale (G.Sc.) vs. Sketch (Sk.) vs. RGB modals. Therefore, $M_{G}$ is retrained with the representation ratio 1:1:3 to regain the performance, and in Figure 12d, $M_{G}$ successfully declines impostors to find match at Rank = 3.

#### 4.3.3. Open-World Scenario

**Figure 14:**
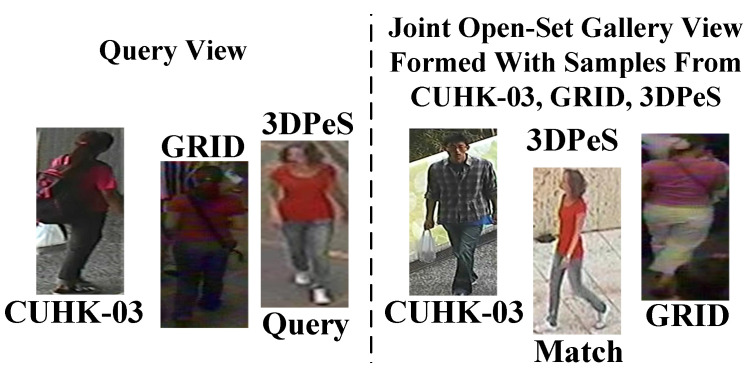
Open-World Scenario where it is not necessary that all the Queries have match in Gallery view. Thus, the metric $M_{G}$ needs to be robust against unknown persons seen in the open world while the target is moved out of perception.

This is the scenario where the target person *P*1 in the real world moves out from the robot perception, and thus the robot losses the target person. While there is no target person in the robot perception, it is required that the robot following the person has robust reidentification capability to resist both impostors in the open world and at the same time has inherent discriminating ability to reidentify the real target person as soon as the target person is recaptured into the robot perception. Therefore, our work also evaluates metric $M_{G}$ in the realistic open world. In Figure 14, we have shown the high-level overview of this scenario.

However, unlike [51], for the open-world testing in our work, randomly, 48 person IDs from each testing domain, i.e., from VIPeR, PRID, GRID, and i-LIDS domains are chosen to form the realistic world joint open-set Gallery, then $M_{G}$ finds the matches for target Query images from the joint open-set Gallery, and the results are given in Table 5. In Table 5, $M_{G}$ in the open world optimizes the part-attention weights in a way to learn cross-representations to simultaneously decline a large number of impostors in the open world and also discriminates difficult nontarget Queries to attain 68.02%, 56.09%, and 76.57% Rank-1 identification at FTR 0.1% on PRID, VIPER, and i-LIDS, respectively.

**Figure 15:**
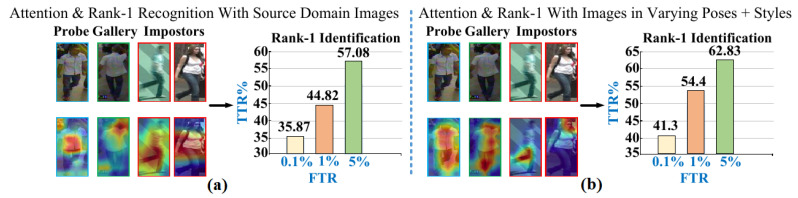
Rank-1 Result Comparison on VIPeR & GRID for (**a**) G.Sc. vs. RGB (**b**) Sk. vs. RGB.

Furthermore, in Figure 15, attention maps and the corresponding rise in Rank-1 identification accuracy are analyzed. In Figure 15b, it is revealed that $M_{G}$ exploits part-attention module and learns cross-representations that declines large number of impostors and nontarget Queries in the open world; thus, Rank-1 accuracy at FTR 0.1% rises to 41.3% from 35.87% in Figure 15a.

#### 4.3.4. Challenging Open-World Scenario

**Figure 16:**
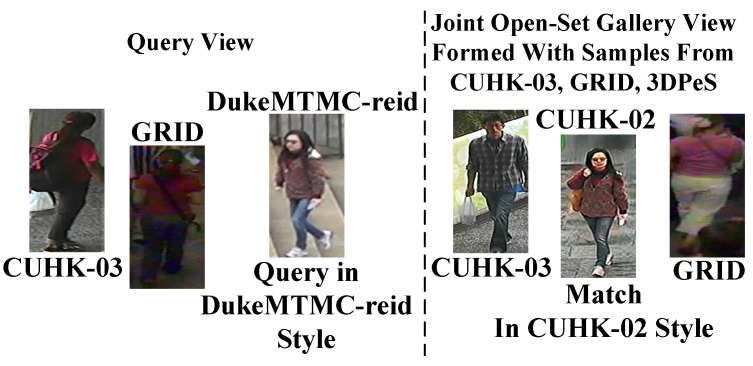
In the Challenging Open-World Scenario, the Query image can be seen in different domains, styles, or modalities, while the match is needed to be found from any other random domain, e.g., the CUHK-02 domain, while the matching image has a style or modality different from that of the Query image.

Though, metric $M_{G}$ is evaluated in the open world; however, open-set Gallery in real-world is far more challenging where Probe-Gallery pairs can be seen in different modals and styles. In Figure 16, this complex scenario is shown visually.

Therefore, images in Query and in joint Gallery are randomly transformed into different modals and styles during testing, then $M_{G}$ finds the matches from the joint multimodal multistyle open-set Gallery, and the results are summarized in Table 6.

$M_{G}$, in contrast to [35,37,38,44], is an open-set metric with inherent ability to match cross-modal and cross-style Probe-Gallery pairs in the nonlinear outdoor environment, while the $M_{G}$ also simultaneously rejects large number of impostors; therefore, in Table 6$M_{G}$ attains 65.11%, 37.51%, 52.08%, and 76.04% Rank-1 identification at FTR 0.1% on PRID, GRID, VIPeR, and i-LIDS datasets, respectively.

**Figure 17:**
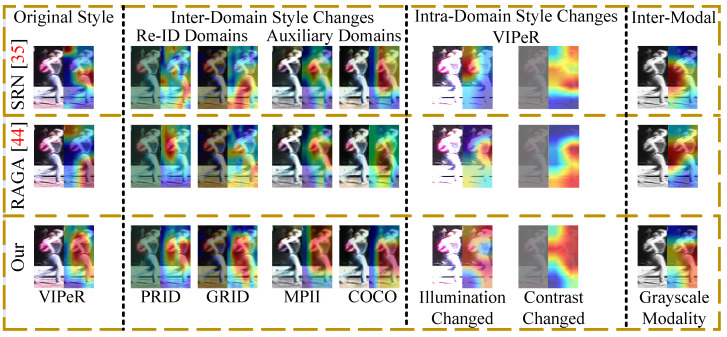
Comparison of Our Part Attention Maps with SRN [35] and RAGA [44].

**Figure 18:**
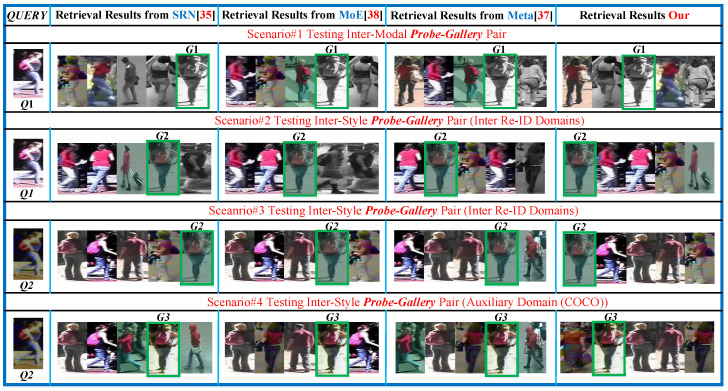
Retrieval results of Our $M_{G}$, SRN [35], Meta [37], and MoE [38] in real-world scenarios. Green Rectangles contain correct matches.

Furthermore, in Figure 17, attention maps and in Figure 18 retrieval results are analyzed in the open-world. Our attention maps in Figure 17 are more focused on individual parts and do not discard unique valuable cues from different filters, therefore, our attention maps are more robust against interenvironment and intraenvironment style and modality transforms than SRN [35]and RAGA [44]. Consequently, $M_{G}$ successfully identifies cross-modal pair (*Q*1,*G*1), and cross-style pairs (*Q*1,*G*2) and (*Q*2,*G*2) at Rank=3, Rank=1, and Rank=1, respectively, in Figure 18; there exists large number of impostors in Gallery in scenario#1, scenario#2, and scenario#3, whereas [35,37,38] inherently lack matching cross-modal and cross-style images. In scenario#4 $M_{G}$, it also matches cross-style pair (*Q*2,*G*3) at Rank=2; even the *G*3 is seen in COCO domain style, where underlying nonlinear transforms and impostors in COCO domain affect the retrieval results.

#### 4.3.5. Cross-Modal Scenario

Our $M_{G}$ is also evaluated against RGB-Infrared matching on SYSU-MM01 dataset. $M_{G}$ compared to [48,54] is trained in real multi-modal open-world to tackle complex nonlinear transforms and can resist large number of impostors; thus, in Table 7, $M_{G}$ attains 64.93% and 72.58% Rank-1 identification on All-search and Indoor-Search, respectively.

#### 4.3.6. Computational Complexity

To run $M_{G}$ in the real world, all convolutions are implemented as mixed depthwise separable convolutions [41]. The computation burden for depthwise separable convolution is K×K×M×H×W + M×N×H×W, whereas the computation burden for standard convolutions are K×K×M×N×H×W. If kernel size K×K is 3×3, and feature map dimensions H×W are 56×56, then, the cost of depthwise separable convolution is 3 × 3 × 256 × 56 × 56 + 256 × 256 × 56 × 56 = 212,746,240 multiplications, while the cost for standard convolutions are 3 × 3 × 56 × 56 × 256 × 256 = 1849,688,064 multiplications. Clearly, depthwise separable convolution lowers the computation cost by 88%, and thus $M_{G}$ is realized on smart embedded camera Hi3516DV300.

#### 4.3.7. Computation Time

Running time of metric $M_{G}$ for different image sizes for one forward pass on Hi3516DV300 are given in Table 8, where for image size 224×224 $M_{G}$ takes 29.4ms to process one single pass and obtain similarity.

### 4.4. Reidentification-Based Tracking Experiments

**Figure 19:**
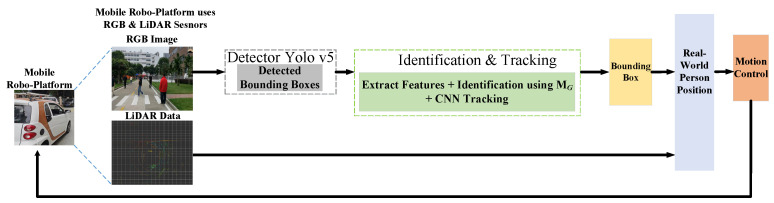
System Overview of Person-Following Robot in Our Work.

To evaluate the learned generic metric $M_{G}$ in the person tracking applications, our work performed several experiments in outdoor to track target. In Figure 19, the complete framework is shown where the learned reidentification metric $M_{G}$ is integrated with pretrained CNN tracker [4].

The input in Figure 19 is RGB image with LiDAR data, where the RGB image is first sent to Yolov5 detector [55] to obtain the bounding boxes. Reidentification module with the tracker in the next step in Figure 19 then takes the detected bounding boxes and identifies the target using the generic metric $M_{G}$ and sends the identified box to the pretrained tracker [4] as the input dynamic template of the person to be tracked. To perform the identification of target, Re-ID metric $M_{G}$ in our work uses prestored features of the target person. Finally, using the tracker prediction box and LiDAR data, the real-world position of the target is then updated. Motion control in last uses the updated position to generate the actuation signals for the robocar.

#### 4.4.1. Experiment Setup and Testing Scenarios

All the experiments in our work are conducted in outdoor, where the person is tracked in different environments, while undergoing illumination and background variations.

**Figure 20:**
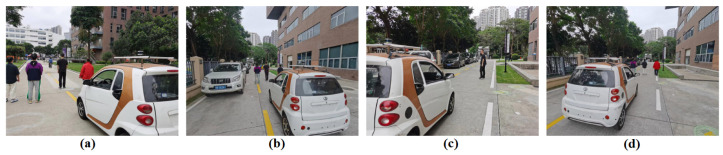
Person-following roboplatform in outdoor world. (**a**) Following the target in black clothing on Road-1; (**b**) Following the target in crowded and occluded scenario on Road-2; (**c**) Continue following the target on Road-3 in varying pose; (**d**) Continue following the target on Road-3 even even there are distractor and impostor.

**Figure 21:**
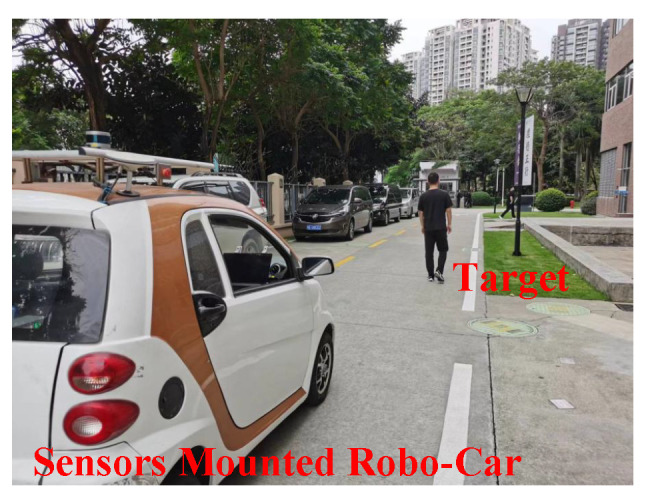
Robocar Robotic Platform mounted with Senors in Outside World.

In Figure 20, it can be seen the person is tracked in three challenging and complex outdoor environments, which are referred to as Scenario#1 (Seq#1), Scenario#2 (Seq#2), and Scenario#3 (Seq#3). In each of these scenarios, the person is tracked for 115 s, 115 s, and 120 s, respectively, while the person in three different scenarios experiences crowding and occlusion with distractors (distractors in our work are the person occluding the target but are the not the impostors of target) and impostors in the scenes, and at several occasions, the person also completely moved out of the perception of mobile roboplatform. The mobile roboplatform visual system consists of RGB camera and LiDAR sensors, both are mounted on top of roboplatform, as shown in Figure 21.

Mounted RGB sensor take images with resolution 1080P with 30 frames per second. Furthermore, the depth and RGB data both are processed with on board CPU i7 8700k on the roboplatform, where the memory size is 16G.

#### 4.4.2. Evaluation Metrics and Comparison

To make fair evaluation and comparison with other state-of-the-art works, our work uses four standard metrics [2] for quantitative evaluation. These metrics both evaluate reidentification performance and tracking success and are defined as: Correctly Identified and Tracked (CT): meaning the person is correctly identified and tracked successfully, Correctly Loss (CL): meaning it is correctly identified the person is not in the scene (either moved out or completely occluded) and thus successfully loss, Wrongly Identified and Tracked (WT): meaning the identification metric wrongly identified the impostor or distractor, and the tracker wrongly tracks the distractor or impostor, and the last metric is Wrongly Loss (WL): meaning the identification metric assumes there is no target in the scene; however, there is target present in the scene, and the tracker wrongly loss tracking.

#### 4.4.3. Results and Analysis

Our work compares the performance with three state-of-the-art trackers, which are STARK [4], DiMP [5], and ATOM [6]. Table 9 below summarizes the results for tracking in all the three scenarios and presents the tracking results for STARK [4], DiMP [5], and ATOM [6], as well as results of our model for the evaluation metrics CT, CL, WT, and WL with the total tracking time in seconds and the percentage of identified and tracked frames in the given Seq#1, Seq#2, and Seq#3.

Results in Seq#1: In Table 9 in Seq#1, our work Correctly Tracked (CT) the target person in 69.6% of total frames, whereas original STARK [4] with no identification module Correctly Tracked (CT) target person in 53.91% of total frames. This shows that tracking when complemented with reidentification module can largely improve the target tracking in different nonlinear scenes.

**Figure 22:**
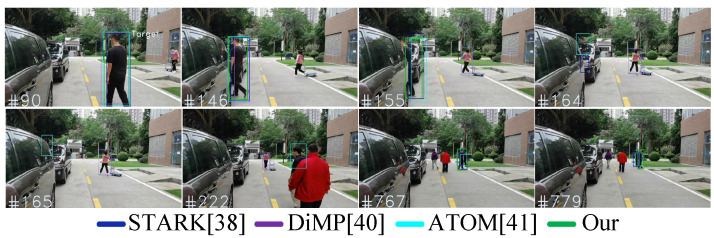
Person-Following Tests in Scenario#1 (seq#1), where the Target moves out of Robocar perception and faces crowding and distractions.

Furthermore, in Figure 22, visual comparison is shown, where it can be seen that when there are no distractors in the frames frame#90 and frame#146, then the identification of target and its tracking is easier; however, when the target is occluded with object in frame#155 in Figure 22, then a few trackers including STARK [4], DiMP [5], and ATOM [6] start tracking distractors. The reason is obvious: STARK [4], DiMP [5], and ATOM [6] all lack the identification ability to distinguish between target and distractor. In addition, when the target is completely moved out of the perception, such as in frames frame#164, frame#165, and frame#222 in Figure 22, then these trackers still continue wrongly tracking either distractors in frame#164 and in frame#165 or impostors in frame#222 since there are no reidentification model to verify if the detected person is a real target or impostor. In last, in Seq#1, it is also evaluated that how the model performs under the scenarios when the person is completely occluded with impostor, such as in frames frame#767 and frame#779. In Figure 22, in frame#767 and frame#779, it can be seen that our model with reidentification metric can successfully identify the person and thereby successfully update the dynamic image template of target to improve tracking, and in last, using both detected bounding box and LiDAR data it can robustly track the person during occlusion. In contrast, in Figure 22, when the person is occluded in frame#767 and frame#779, then DiMP [5] and ATOM [6] wrongly start tracking the impostor in frames frame#767 and frame#779.

Results in Seq#2: In Table 9 in Seq#2, our work Correctly Tracked (CT) the target person in 63.48% of total frames, whereas original STARK [4] with no identification module Correctly Tracked (CT) target person in 48.69% of total frames.

**Figure 23:**
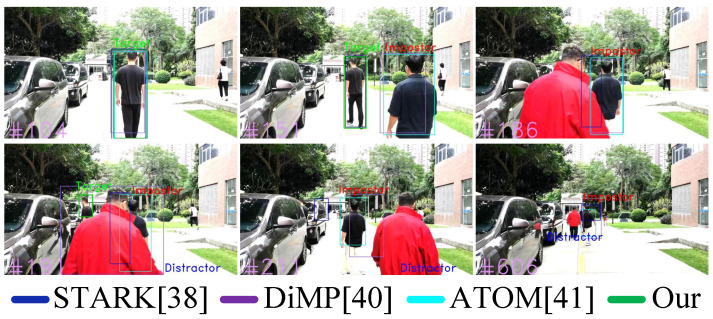
Person-Following Tests in Scenario#2 (seq#2), where the Target moves in varying illumination environment, and is occluded with impostors and distractors, while, Target also moves out of perception.

The results are lower than in Seq#1 because Seq#2 is more challenging than Seq#1, where in Seq#2 person moves in a varying illumination environment, while the background noise also affect reidentification, as shown in Figure 23. Furthermore, in Figure 23, the person is also occluded by distractor and impostor in the scene. In Seq#2 in frame#151 and frame#186, our reidentification metric $M_{G}$ can identify the target successfully in the presence of impostor while other trackers DiMP [5] and ATOM [6] follow impostor person. Furthermore, in frame#191 in Figure 23, when both impostor and target are seen in the scene, the target is occluded by both impostor and distractor; then, in such a scenario, still our identification metric $M_{G}$ can address both occlusion and illumination variation to identify the target and thereby improve its tracking. Similarly, in other complex scenes, when target is completely moved out of perception in frame#211 in Figure 23 and when the target is occluded by both impostor and distractor in frame#606 in Figure 23, then, in both scenes, our reidentification metric $M_{G}$ helps tracker to not follow impostor, and the mobile roboplatform stops and wait for the target to reappear and identified.

**Figure 24:**
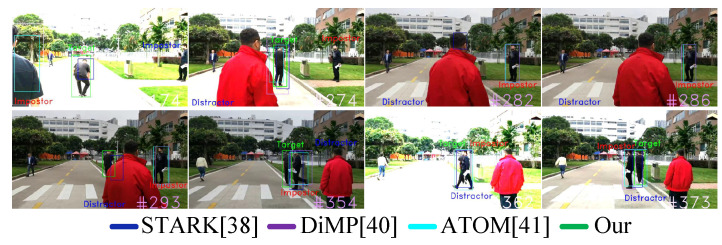
Person-Following Tests in Scenario#3 (seq#3), where the Target moves in varying illumination environment, while the Target is completely occluded with impostors and distractors, and also suffers due to changing backgrounds.

Results in Seq#3: In Table 9 in Seq#3, our work Correctly Tracked (CT) the target person in 60.02% of total frames, whereas original STARK [4] with no identification module Correctly Tracked (CT) target person in 34.78% of total frames. Seq#3 is far more challenging than Seq#2 and Seq#1, where in Seq#3 the person simultaneously undergoes pose and viewpoint changes, illumination and background variations, as well as, distracted and occluded by distractors and impostors, as shown in Figure 24. However, still, our reidentification metric $M_{G}$ successfully identified and tracked the person in 60.02% of total frames, compared to the state of art STARK [4], DiMP [5] and ATOM [6]. This is mainly due to the correct identification of the target, which helps the tracking. In Figure 24, in frame#174 in Seq#3, the target person undergoing varying posture, while impostors are in the seen; however, still the learned generic metric $M_{G}$ discriminates the target well. Furthermore, in frame#282 and frame#286 in Figure 24, when the target is fully occluded by distractor, while an impostor is nearby, still $M_{G}$ successfully discriminates the impostor, whereas DiMP [5] and ATOM [6] track the impostors. In addition, in frame#293 in Figure 24, when the target reappears after full occlusion, then both our model and STARK [4] track the target well; however, STARK [4] has a little higher localization error in tracking than our model.

Last, in Figure 24 in frames frame#354 and frame#362, the target again occluded by impostor, while, also undergo illumination variations. Though, the situation is challenging, but the metric $M_{G}$ is trained to address both illumination variations and style variations, and it is resistant against impostors; therefore, $M_{G}$ continues identifying the person in consecutive frames, i.e., in frame#354, frame#362, and in frame#373.

## 5. Conclusions & Future Directions

This work learns lightweight domain generic metric in the multimodal open world for person-following robots to address the practical world challenges face by person-following robots including nonlinear pose, viewpoint, style, and multimodal transforms, and a novel part-attention module is proposed to learn attention weighted cross-representations to address displacement and occlusion of parts. Thereby, the learned generic metric can resist large number of impostors and nontarget queries in the open world, while the learned metric is also lightweight and can run on robotic platform. Furthermore, future research will focus to improve the learned domain generic reidentification metric to solve multiscale and night reidentification problems for person-following robots.

## Figures and Tables

**Table 1:** Detail of each layer, here, DW: Depthwise and MDW: Mixed-Depthwise. All different convolutions in MDW are 1-layer.

Layer Name	Output Size	10-Layer
conv1	112 × 112	7 × 7 (DW-Conv.), 64, stride 2
conv2_x	56 × 56	3 × 3, Max Pool, stride 2
		3 × 3 (DW-Conv.), 128
conv3_x	28 × 28	$\left[\begin{array}{c} 3\;\times \;3,\;5\;\times \;5\;(\mathrm{MDW}-\mathrm{Conv}.),\;256\\ 3\;\times \;3,\;5\;\times \;5,\;7\;\times \;7\;(\mathrm{MDW}-\mathrm{Conv}.),\;256 \end{array}\right]$ × 1
conv4_x	14 × 14	$\left[\begin{array}{c} 3\;\times \;3,\;5\;\times \;5\;(\mathrm{MDW}-\mathrm{Conv}.),\;512\\ 3\;\times \;3,\;5\;\times \;5,\;7\;\times \;7\;(\mathrm{MDW}-\mathrm{Conv}.),\;512 \end{array}\right]$ × 1
conv5_x	7 × 7	$\left[\begin{array}{c} 3\;\times \;3,\;5\;\times \;5\;(\mathrm{MDW}-\mathrm{Conv}.),\;1024\\ 3\;\times \;3,\;5\;\times \;5,\;7\;\times \;7\;(\mathrm{MDW}-\mathrm{Conv}.),\;1024 \end{array}\right]$ × 1
	1 × 1	Global Average Pool, 512-d FC1, 256-*d* FC2, Softmax
FLOPs		1.1 $\times \;10^{9}$

**Table 2:** Results on Naïve Close-Set Setting#1.

Methods	Target:PRID		Target:GRID		Target:VIPeR		Target:i-LIDS	
	Rank-1	mAP	Rank-1	mAP	Rank-1	mAP	Rank-1	mAP
CBAM [45]	47.52	53.89	33.07	39.17	45.80	51.87	75.08	81.0
HLGAT [52]	48.74	56.84	35.22	43.94	48.96	58.03	79.67	82.64
RAGA [44]	50.69	60.47	37.79	45.51	49.03	58.33	83.91	85.2
SRN [35]	52.1	66.5	40.2	47.7	52.9	61.3	84.1	89.9
D.Norm [36]	60.4	-	41.1	-	53.9	-	74.8	-
MoE [38]	57.7	67.3	46.8	54.2	56.6	64.6	85.0	90.2
Meta [37]	74.2	81.0	48.4	57.9	59.9	68.6	81.3	87.0
Our $M_{G}$	80.73	86.91	55.07	61.23	64.44	75.1	88.99	91.0

**Table 3:** Results on Naïve Close-Set Setting#2.

Methods	Target:PRID		Target:GRID		Target:VIPeR		Target:i-LIDS	
	Rank-1	mAP	Rank-1	mAP	Rank-1	mAP	Rank-1	mAP
CBAM [45]	39.42	51.33	25.21	37.99	32.61	44.04	69.24	73.0
HLGAT [52]	43.95	50.07	28.77	39.5	37.66	49.72	70.77	76.95
RAGA [44]	44.49	50.1	30.92	40.32	38.55	51.79	72.14	78.63
SRN [35]	47.85	51.72	34.87	43.67	44.99	52.05	72.1	78.41
D.Norm [36]	53.70	60.1	37.22	44.33	49.54	54.41	71.04	80.03
MoE [38]	51.51	63.91	39.35	47.69	48.74	57.0	79.0	83.1
Meta [37]	70.82	78.07	42.61	50.95	51.74	59.97	79.68	84.78
Our $M_{G}$	77.89	84.78	52.64	58.85	61.92	73.08	86.23	90.0

**Table 4:** Results on Challenging Close-Set Scenario.

Methods	Target:PRID		Target:GRID		Target:VIPeR		Target:i-LIDS	
	Rank-1	mAP	Rank-1	mAP	Rank-1	mAP	Rank-1	mAP
CBAM [45]	30.98	40.88	21.8	28.4	25.74	32.37	58.37	62.66
D.Norm [36]	31.49	42.41	23.54	30.68	27.73	35.20	60.56	69.03
RAGA [44]	34.93	42.99	24.12	32.34	32.64	37.85	64.05	73.08
HLGAT [52]	36.79	43.35	24.31	33.14	35.25	46.39	65.43	76.33
SRN [35]	40.23	49.43	25.06	34.84	38.98	50.27	70.47	76.23
MoE [38]	45.12	53.33	28.49	37.19	44.46	57.96	75.64	80.27
Meta [37]	62.85	71.33	30.94	39.0	44.05	57.37	71.01	79.95
Our $M_{G}$	75.04	82.19	49.38	57.83	60.17	71.93	84.04	88.13

**Table 5:** Results on Open-World Re-ID.

Methods	Target:PRID		Target:GRID		Target:VIPeR		Target:i-LIDS	
	Rank-1	mAP	Rank-1	mAP	Rank-1	mAP	Rank-1	mAP
CBAM [45]	26.95	51.00	18.43	34.26	23.48	44.38	41.07	68.98
APN [51]	28.63	53.29	19.34	36.60	24.80	46.21	43.32	70.87
RAGA [44]	31.05	55.74	19.92	38.34	27.42	49.49	45.82	73.03
HLGAT [52]	32.98	57.06	20.27	40.97	28.14	51.64	47.12	75.55
SRN [35]	37.34	60.49	23.13	44.17	30.43	53.42	50.38	77.00
MoE [38]	43.01	65.09	26.36	47.15	33.01	56.10	52.18	80.67
Meta [37]	45.70	65.39	28.43	49.68	32.11	55.27	51.35	80.29
Our $M_{G}$	68.02	80.86	41.3	54.4	56.09	78.1	76.57	86.42

**Table 6:** Results on Challenging Open-World Re-ID.

Methods	Target:PRID		Target:GRID		Target:VIPeR		Target:i-LIDS	
	Rank-1	mAP	Rank-1	mAP	Rank-1	mAP	Rank-1	mAP
CBAM [45]	19.34	38.27	10.66	20.03	17.58	33.71	28.0	52.14
APN [51]	21.01	47.75	12.12	25.34	18.97	36.40	32.31	59.86
RAGA [44]	22.65	49.38	12.23	25.96	19.72	38.04	34.54	60.43
HLGAT [52]	23.54	51.03	14.18	28.81	21.17	40.47	36.84	64.46
SRN [35]	26.09	54.80	16.83	34.34	23.24	43.22	39.13	68.08
MoE [38]	29.37	59.0	19.04	38.71	25.79	48.35	42.86	71.53
Meta [37]	32.45	60.07	21.22	40.48	25.35	47.98	42.24	71.22
Our $M_{G}$	65.11	80.34	37.51	51.77	52.08	76.9	76.04	86.0

**Table 7:** Cross-Modal Comparison on SYSU-MM01.

Methods	SYSU-MM01					
	All-Search			Indoor-Search		
	R = 1	R = 10	mAP	R = 1	R = 10	mAP
Hi-CMD [53]	34.94	77.58	35.94	-	-	-
GECNet [48]	53.37	89.86	51.83	60.60	94.29	62.89
cm-SSFT [54]	61.6	89.2	63.2	70.5	94.9	72.6
Our $M_{G}$	64.93	92.31	66.04	72.58	94.89	72.98

**Table 8:** Computation Time for Different Image Sizes.

Image Size	Time/Image (ms)	Device Platform
324×324	52	Hi3516DV300
256×256	38.4	Hi3516DV300
224×224	29.4	Hi3516DV300

**Table 9:** Person Reidentification Results for Tracking in the Outdoor World.

		Methods							
		DiMP [5]		ATOM [6]		STARK [4]		Ours	
		Time (Sec)	Frames (%)	Time (Sec)	Frames (%)	Time (Sec)	Frames (%)	Time (Sec)	Frames (%)
Seq. #1	CT	52	45.2	57	49.57	62	53.91	80	69.6
	CL	20	17.39	17	14.78	16	13.92	25	21.74
	WT	13	11.29	15	13.05	13	11.31	6	5.21
	WL	30	26.09	26	22.61	24	20.87	4	3.48
Seq. #2	CT	31	26.96	44	38.26	56	48.69	73	63.48
	CL	17	14.78	10	8.69	12	10.43	25	21.74
	WT	22	19.13	19	16.52	15	13.04	8	6.96
	WL	45	39.13	42	27.83	32	26.08	9	7.82
Seq. #3	CT	28	24.35	38	33.04	40	34.78	69	60.02
	CL	14	12.17	8	6.95	10	8.69	29	25.22
	WT	26	22.61	23	20	20	17.39	12	10.43
	WL	52	45.22	51	44.35	50	43.48	9	7.83

## Data Availability

Not applicable.
